# Flavonoids—Natural Gifts to Promote Health and Longevity

**DOI:** 10.3390/ijms23042176

**Published:** 2022-02-16

**Authors:** Xiaolan Fan, Ziqiang Fan, Ziyue Yang, Tiantian Huang, Yingdong Tong, Deying Yang, Xueping Mao, Mingyao Yang

**Affiliations:** 1 Institute of Animal Genetics and Breeding, Sichuan Agricultural University, Chengdu 611130, China; xiaolanfan@sicau.edu.cn (X.F.); f18717044553@163.com (Z.F.); ziyueyang2022@163.com (Z.Y.); huangtiantian1666@163.com (T.H.); tyd5786195@126.com (Y.T.); dnaydy@126.com (D.Y.); mxp2020@sina.com (X.M.); 2 Farm Animal Genetic Resources Exploration and Innovation Key Laboratory of Sichuan Province, Sichuan Agricultural University, Chengdu 611130, China

**Keywords:** flavonoids, macromolecular damage, health span, aging

## Abstract

The aging of mammals is accompanied by the progressive atrophy of tissues and organs and the accumulation of random damage to macromolecular DNA, protein, and lipids. Flavonoids have excellent antioxidant, anti-inflammatory, and neuroprotective effects. Recent studies have shown that flavonoids can delay aging and prolong a healthy lifespan by eliminating senescent cells, inhibiting senescence-related secretion phenotypes (SASPs), and maintaining metabolic homeostasis. However, only a few systematic studies have described flavonoids in clinical treatment for anti-aging, which needs to be explored further. This review first highlights the association between aging and macromolecular damage. Then, we discuss advances in the role of flavonoid molecules in prolonging the health span and lifespan of organisms. This study may provide crucial information for drug design and developmental and clinical applications based on flavonoids.

## 1. Introduction

Aging is thought to be one of the risk factors for chronic diseases responsible for the most morbidity, mortality, and health care consumption worldwide [1,2]. Such chronic diseases include atherosclerosis, cardiovascular disease, stroke, most cancers, diabetes, kidney failure, chronic lung disease, osteoporosis, arthritis, blindness, dementia, and neurodegenerative diseases. Aging will also make people prone to geriatric syndrome and to a decline in immunity and physical recovery. These chronic diseases often occur in older individuals. By understanding how aging enables pathology, new therapeutics will arise for multiple chronic diseases, providing an opportunity to extend the human health span by targeting aging directly [3]. Therefore, finding anti-aging drugs that meet the safety and effectiveness of long-term use has always been an important strategy for intervention in the aging field.

**Figure 1:**
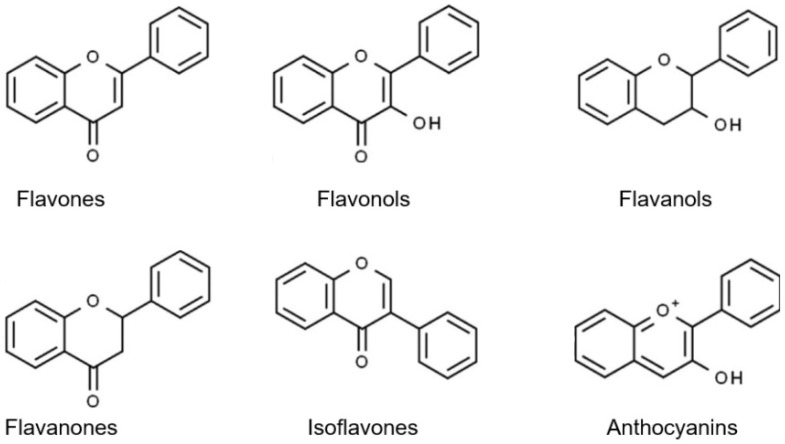
Basic structures of the major naturally occurring flavonoids.

Flavonoids are a diverse family of natural phenolic compounds commonly found in fruits, vegetables, tea, wine, and Chinese herbal medicine [4]. Flavonoids have a basic C6–C3–C6 15 carbon skeleton composed of two aromatic rings and one pyran ring. Flavonoid compounds are divided into six subclasses based on their carbon structure and level of oxidation, which are flavones, flavonols, flavanones, isoflavones, flavanol, and anthocyanins (Figure 1) [5]. In addition to the well-known antioxidant activity, flavonoids also possess anti-inflammatory, vasodilator, anticoagulant, cardioprotective, antidiabetic, chemical protection, neuroprotective, and anti-obesity activities [5]. Recent studies have shown that flavonoids also have suitable anti-aging activities. The combination of quercetin and dasatinib has been observed to eliminate senescent cells in vitro, improve physical function, and increase the lifespan of mice in vivo [6]. More interestingly, in phase I clinical trials in patients with diabetic kidney disease [7] and idiopathic lung disease [8], dasatinib administration with quercetin has been shown to effectively reduce the expression of the aging markers p16 and SA-β-gal. More flavonoids, such as fisetin and luteolin, have also been found to eliminate senescent cells and have anti-aging effects [9,10]. However, the anti-aging mechanism of flavonoids is not yet fully understood, and more research is needed to provide a basis for their clinical applications in humans.

Here, we summarize the latest research progress on flavonoids with anti-aging benefits. Particular attention is given to their effect on delaying the accumulation of unrepaired damage in the cell by reducing the harm caused by macromolecules or enhancing the repairability of the cell. The role of flavonoids in preclinical and clinical aspects is also discussed. This has the potential to provide necessary information for the design and development of drugs based on these compounds and the clinical use of anti-aging agents.

## 2. Cellular Senescence Is Driven by Unrepaired Damage

**Figure 2:**
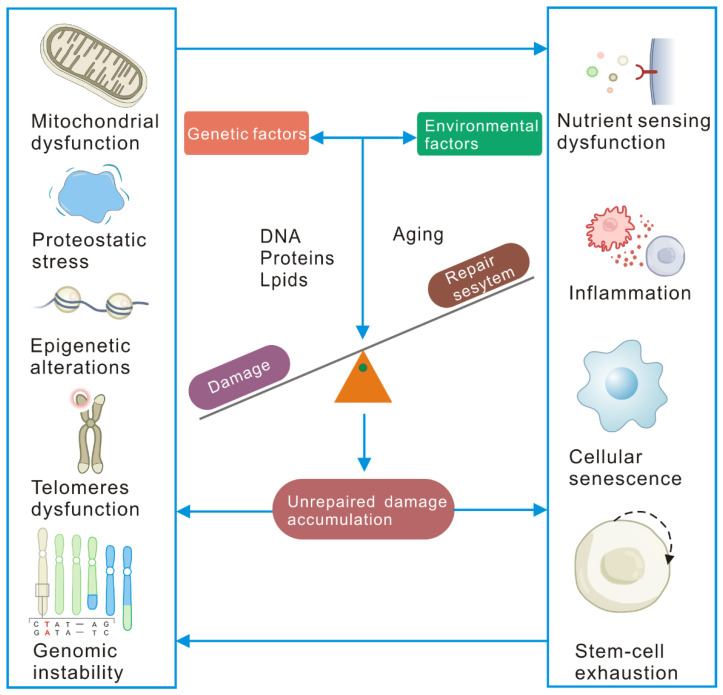
Diagram of the major influences and mechanisms by which macromolecular damage induces aging. Damage insults (genotoxic stress, oxidative stress, etc.) in genetic or environmental factors damage macromolecules (mainly including DNA, proteins, and lipids) during the aging process, causing intracellular damage to accumulate. At the same time, the repairability in the cell declines with aging, which causes the accumulation of unrepaired damage in the cell. Accumulated unrepaired damage can lead to mutations or chromosomal aberrations, leading to genome instability. Severely shortened telomeres activate the DNA repair and damage response (DDR) and cause cell senescence. Accumulated unrepaired damage affects autophagy and the ER-UPR and results in the loss of protein complex stoichiometry. Mitochondrial dysfunction is driven by NAD+ deprivation caused by nuclear DNA repair, mitochondrial autophagy defects induced by DNA damage, and changes in the expression of mtDNA polymerase that affect mtDNA replication. The accumulated unrepaired damage wreck the nutrient-sensing pathway, affecting repair and signal transduction. The accumulated unrepaired damage induces cell senescence and leads to the exhaustion of the stem cell pool through DDR-induced apoptosis, senescence, premature differentiation, and changes in the niche of stem cells. Cell senescence affects cell-to-cell communication through inflammatory cytokines and inhibitory growth signals.

Although the current understanding of aging is still in the early stages of genetic discovery, existing evidence shows that human aging is driven by the balance of damage and repair processes and is affected by environmental exposure and genetics (Figure 2). One of the characteristics of aging is its association with macromolecular damage. When the organism cannot replace cells at will or dilute the damage, intracellular damage accumulates, hurting the host cell and other cells, impairing its function and ultimately leading to age-related diseases and aging itself. The nine hallmarks of aging have been summarized [2] and are widely recognized by aging research scientists. Genomic instability, telomere attrition, epigenetic alterations, and loss of proteostasis are the primary causes of damage. The most common types of macromolecular damage are DNA protein and lipid damage.

### 2.1. DNA Damage and Repair

DNA damage has been thought to be a strong candidate as the primary cause of aging [11]. DNA damage includes oxidative modifications, single- and double-strand breaks (DSBs), and mutations, both in vitro and in vivo [12,13]. Many studies have indicated that DNA damage accumulation is associated with aging [14,15]. A complete DNA repair system is also established to repair DNA damage in cells. Prominent DNA repair pathways in mammalian cells are base excision repair (BER), mismatch repair (MMR), nucleotide excision repair (NER), and double-strand break repair (DSBR). It has been observed that the ability to repair DNA damage decreases with aging [16]. Thus, unrepaired DNA damage further accumulates during aging. Unrepaired DNA damage can cause genome instability and induce a signal cascade that leads to cell senescence or death and related cell aging phenotypes [17,18]. More than 50 DNA repair disorders have been described as having varying degrees of overlapping phenotypes with aging, such as neurodegeneration, cancer, and cardiovascular disease [19].

### 2.2. Protein Damage

Various internal and external factors constantly damage intracellular proteins. Damage to proteins, in turn, may affect myriad intracellular pathways given their abundance. Protein quality control (PQC) is critical to maintaining a functioning proteome. The quality of the protein is guaranteed by the translation mechanism and the activity of auxiliary proteins (including molecular chaperones), while degradation is controlled by autophagy and proteasome functions. The accumulation of protein damage in the aging process is mainly due to (i) decreased translation fidelity [20,21], (ii) downregulation of protein chaperones [22,23], and (iii) decreased proteasome activity [24] and other factors in protein synthesis and quality control. Damaged proteins contribute to proteostatic stress, the accumulation of misfolded/aggregated proteins, and protein toxicity, which further aggravate the senescence of cells.

### 2.3. Lipid Damage

Lipid damage is mainly due to lipofuscin, a nondegradable protein and lipid oxidation product, which accumulates in senescent cells [25]. Lipofuscin is an autofluorescent lipopigment formed by lipids, metals, and misfolded proteins, which is especially abundant in nerve cells, cardiac muscle cells, and skin [26]. Lipofuscin is emerging as another indicator of senescent cells in culture and in vivo [27,28]. Recent research results indicate that lipofuscin can actively change cell metabolism, cell death, and apoptosis at different levels by inhibiting proteasomes, weakening autophagy and lysosomal degradation, and acting as a metal ion pool to cause ROS generation [29]. In addition, the dispersive nature of the deposits distributed throughout the tissue may support the mechanism of lipofuscin diffusion and seeding of new lipofuscin aggregates [30]. It should be noted that damage accumulation continues even when cell division ceases and can continue for months or even years.

### 2.4. Molecular, Cellular, and Systemic Consequences of Unrepaired Damage Accumulation

When damage accumulates, it will drive cell fate decisions and aging-related events. Unrepaired damage is closely related to molecular consequences such as genome stability, dysfunctional telomeres, epigenetic alterations, protein homeostasis, and intracellular mitochondrial dysfunction during aging. Accumulating evidence suggests that DNA damage is a significant driver of age-associated epigenetic changes [31,32]. DNA damage may cause protein homeostasis stress by increasing transcriptional arrest (transcriptional pressure) or transcription noise mediated by mutations or epimutations. This may affect the assembly, stoichiometry, correct folding, and function of proteins and protein complexes, leading to steady-state protein stress and aggregation. Age-related motor dysfunction and damaged mitochondrial pathology were found in E3 ubiquitin ligase *parkin*-deficient mice, indicating that impaired mitochondrial clearance caused by *parkin* deficiency may be the basis of Parkinson’s disease pathology [33]. DNA damage repair itself can strain the protein homeostasis mechanism [34]. Vegetarian food containing lipofuscin reduces the athletic performance of young fruit flies, and the accumulation of AGE-modified protein and carbonylation protein in the somatic tissues and the hemolymph is accelerated, significantly reducing the health span of fruit flies [35].

Damage at the cellular level drives cell senescence and exhausts stem cell pools (Figure 2). Compounds such as bleomycin, doxorubicin, or cisplatin often cause irreparable DNA damage and drive cell senescence [36]. The translation error significantly increased in aging flies, while increased fidelity of protein synthesis extended the lifespan across species [21]. Lipofuscin has been reported as a hallmark of senescent cells [37]. The accumulation of damage in tissues can also affect the microenvironment in the stem cell niche or the systemic circulation of factors that affect the aging of stem cells and organs. There have been reports of the accumulation of age-related DNA damage in elderly *Drosophila* intestinal stem cells [38] and intestinal crypts of mice [39]. Thus, damage to the cells accelerates the senescence of cells and stem cells.

The damage accumulation also affects the immune microenvironment and nutrient sensing in aging. In *C. elegans*, DNA damage can trigger an innate immune response, enhancing proteostasis and systemic stress resistance [40]. The chaperone protein HSP70 acts as a bridge between the ubiquitin E3 ligase PDLIM2 and the proteasome to inhibit proinflammatory NF-κB signaling [41]. Damage and repair systems regulate nutrient-sensing pathways, including ILS, sirtuins, and AMP-activated protein kinase (AMPK)-regulated mTOR pathways [42,43]. The DNA damage sensor ATM can activate the AMPK pathway in response to energy changes. mTOR itself is transiently phosphorylated following DNA damage in an ATR (damage sensor)-dependent manner [44]. The ubiquitin ligase complex GID regulates AMPK activity and organismal lifespan [45].

In summary, the accumulation of damage is one of the leading causes of cellular senescence, the systemic imbalance between cells, and the primary hallmarks of senescence.

## 3. Flavonoid Compounds Serve as Anti-Aging Agents

Over the last two decades, flavonoids have drawn attention as promising natural dietary molecules to prevent aging and aging-related diseases. According to their different ways of interfering with aging, anti-aging flavonoids are divided into senolytic flavonoids, senomorphic flavonoids, and antisenescence activity (Table 1).

### 3.1. Senolytic Flavonoids

Senescent cells and the senescence-related secretion phenotypes (SASPs) secreted by them are essential factors leading to the aging of tissues and organs [6]. Therefore, therapeutic approaches to specifically kill senescent cells can extend health span and lifespan. “Senolytic” compounds can kill senescent cells [75]. Quercetin is effective against senescent human endothelial cells in combination with dasatinib, which is more effective in eliminating senescent MEFs [46], reducing the expression of SASP factors [47]. Moreover, quercetin plus dasatinib has been proven to enhance health span and lifespan in old mice [6] and improve age-related diseases such as cardiovascular disease and temporomandibular joint degeneration [76]. Furthermore, in an open-label clinical trial, within three weeks, oral quercetin and dasatinib improved the 6-min walking distance, walking speed, and ability to stand up from a chair and shortened the body function battery five days after the last dose [5,77].

In a panel of 10 polyphenols examined, fisetin was potently senolytic in cultured senescent murine and human fibroblasts, while luteolin had a weak effect on clearing senescent cells. Fisetin increased the median and maximum lifespans of aged mice [9]. Notably, fisetin treatment significantly reduced mortality, cellular senescence, and inflammatory markers and increased antiviral antibodies when the SARS-CoV-2-related mouse β-coronavirus was exposed to old mouse pathogens [78]. As fisetin has a good effect against inflammatory factors, it has been used in clinical research to alleviate the dysfunction of COVID-19 and the excessive inflammatory response in the elderly (NCT04537299). Burton et al. showed that luteolin significantly reduced the proportion of microglia stained for IL-1β and IL-6 in LPS-treated adult mice [10].

### 3.2. Senomorphic Flavonoids

Senomorphics refer to compounds and dietary supplements that can restrain senescence-associated phenotypes by explicitly suppressing the SASP or proinflammatory secretome. Recent research results also show that the flavonoids apigenin, kaempferol, and 4,4′dimethoxychalcone also have such “senomorphic” effects (Table 1). Apigenin belongs to the flavone subclass of flavonoids and can delay the aging process by activating the Nrf2 pathway [79]. Apigenin partially inhibits SASP by inhibiting IL-1α signaling in human fibroblast cell lines through IRAK1 and IRAK4, p38-MAPK, and NF-κB [49]. Kaempferol is a flavonol, and it significantly inhibited IL-6, IL-8, and IL-1b expression but did not considerably affect senescence itself in bleomycin-induced senescent BJ cells. A cellular mechanism study showed that kaempferol in senescent BJ cells might be mediated, at least in part, by interfering with IRAK1/IkBa/NF-kB p65 signaling [50,80].

### 3.3. Another Antisenescence Activity of Flavonoids

In addition, an increasing number of flavonoids have been proven to delay the aging process. As shown in Table 1, these compounds include various subsets of flavonoids. The flavonoid 4,4′-dimethoxychalcone (DMC) is derived from *Angelica keiskei koidzumi*, a plant with longevity- and health-promoting effects in traditional Chinese medicine. DMC extends the lifespan of yeast, worms, and flies and decelerates the senescence of human cell cultures via GATA transcription factors to induce autophagy [51].

Naringenin and nobiletin are widely found in the fruits of *Citrus* L. plants in the Rutaceae family. Both of them have antioxidant effects and can reduce ROS in senescent cells. In addition, naringenin has a significant impact on reducing cardiovascular markers of damage caused by aging [52]. The lifespan analysis experiment in *Drosophila* showed that treatment with 400 µm/L of naringenin could prolong lifespan by up to 22.62% [53]. However, nobiletin’s role is mainly in regulating abnormal energy metabolism. Nobiletin targets retinoid acid receptor-related orphan receptors (RORs) to remodel circadian and metabolic gene expression, enhancing the circadian rhythm and preventing metabolic syndrome [66]. Furthermore, nobiletin-RORs have been reported to optimize skeletal muscle mitochondrial respiration and promote healthy aging in high-fat diet mice [67].

Genistein is an isoflavone derived from soy products. Genistein induces autophagy to reduce cell senescence in vascular smooth muscle cells [55]. Genistein reduced age-related increases in NF-κB activity and NF-κB-dependent proinflammatory gene expression in vivo in rats; thus, it can be used as an anti-inflammatory compound [56]. Antisenescence effects have also been reported for epicatechin. Epicatechin induces the reversal of endothelial cell senescence and improves vascular function in rats [63]. Supplementation with epicatechin has been observed to improve the survival rate of elderly mice and age-related phenotypes such as skeletal muscle degeneration [64] and brain dysfunction [65].

Myricetin and dihydromyricetin are produced in several plants, particularly in some commonly consumed fruits and vegetables (strawberries, grapes). They have been approved as food supplements in Europe and the United States. Survival experiments show that both compounds prolong lifespan [58,60]. Interestingly, myricetin and dihydromyricetin have been reported to have anti-AD effects [81].

Rutin, a natural flavonoid glycoside compound, has revealed an extensive anti-aging effect. Rutin can induce autophagy to extend the lifespan of *Drosophila* treated with HDF [68] and can also effectively improve the metabolic dysfunction associated with aging by regulating the IIS signaling pathway [69]. Moreover, the administration of rutin reduces the expression of ROS and proinflammatory cytokines (TNF-α and IL-1β) in neuronal cells, which can prevent the development of AD and protect the aging brain or slow the neurodegenerative process [70].

Hesperidin is a flavanone glycoside derived from citrus that has been found to possess various pharmacological properties including antioxidant, cholesterol-lowering, and anti-inflammatory ones. Topical application of hesperidin can improve functional abnormalities of the aging epidermis including abnormal epidermal permeability barrier function, epidermal differentiation, lipid production, and stratum corneum acidification [82]. Hesperidin upregulated Nrf2 and reduced ROS, significantly prolonging the replicative lifespan of yeast [71]. Hesperidin treatment also effectively protected the hearts of aged rats by upregulating the protein level of Nrf2 and increasing the activity of enzymatic antioxidants [72]. In addition, some other citrus flavonoids such as naringin, hesperitin, and neohesperidin have also maintained ROS scavenging and potential anti-aging activities in yeast [83].

Theaflavins are derived from the conversion of catechins by endogenous polyphenol oxidase and peroxidase during the production of black tea [84]. Studies have shown that theaflavin can delay the excessive proliferation of intestinal stem cells, prevent intestinal dysbiosis, and inhibit the activation of the Imd signaling pathway, thereby prolonging the lifespan of *Drosophila*. At the same time, theaflavin is effective in preventing DSS-induced colitis in mice [73]. Moreover, theaflavin can protect against oxidative stress-induced cellular senescence by activating Nrf2 in a mouse osteoarthritis model [85]. Furthermore, treatment of middle-aged mice with theaflavin 3-gallate reduced senescence in hypothalamic neural stem cells while improving senescence-related pathology [74].

In short, flavonoids with anti-aging effects are diverse in both their types and their modes of action. Molecules of the same subclass also have anti-aging targets, showing that more detailed research is needed to reveal their respective regulatory mechanisms.

## 4. Benefits of Flavonoids in Attenuating Aging Damage

**Figure 3:**
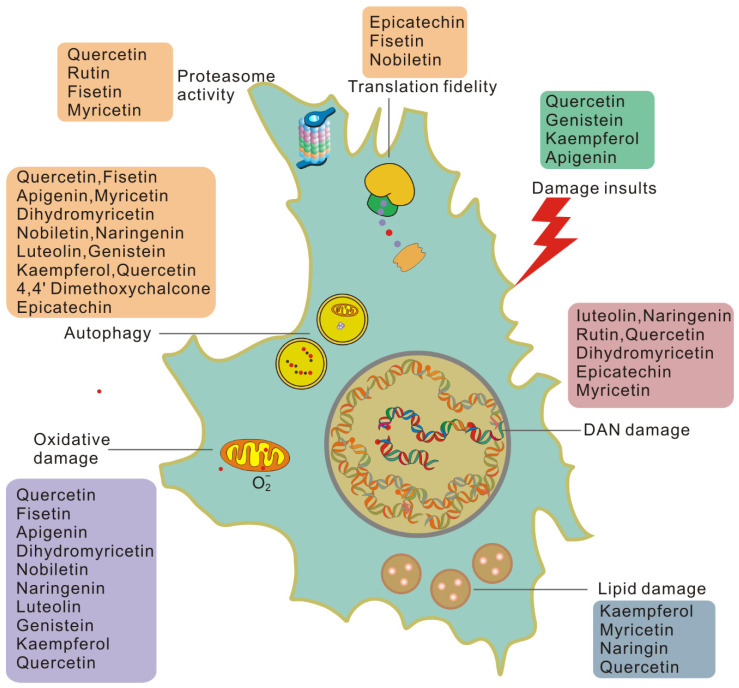
Flavonoids work on each type of damage-dependent trigger of cellular senescence. Cells induced to senesce by damaging insults exhibit higher basal levels of damage than healthy cells and generate damage at a higher rate.

Due to the important impact of damage on cellular and systemic aging, the removal or repair of damage will help re-establish the equilibrium state of damage repair and, thus, slow down the aging rate. Many findings suggest that flavonoids play an essential role in reducing damage and rebuilding tissue homeostasis, as shown in Figure 3.

Flavonoids can reduce cellular damage caused by a variety of damage insults. Quercetin protects red blood cells from oxidative stress and genotoxicity in vitro [86]. Quercetin can also protect cells from the stress of misfolded proteins in the endoplasmic reticulum [87]. Genistein may significantly reverse the misfolding of the N-CoR protein induced by PML-RAR by inhibiting the selective phosphorylation-dependent binding of N-CoR and PML-RAR [88]. Kaempferol [89] and apigenin [90] may alter the protein associated with the internal ribosome entry site (IRES) to limit viral infection and inhibit viral IRES-driven translation activities. In this way, flavonoids can reduce cell damage from the source.

Many flavonoids can act on DNA damage in a variety of ways. The flavonoids luteolin, naringenin, and rutin effectively attenuate UVB-induced DNA damage in vitro [91] and in vivo [92]. Quercetin has been reported to effectively reverse 1,2-dimethylhydrazine-mediated oxidative stress and DNA damage by targeting the NRF2/Keap1 signaling pathway in rats [93]. Recently, nanocapsules containing dihydromyricetin were reported to have a 50% sun protection factor (SPF-DNA) against DNA damage caused by UVB radiation and 99.9% protection against DNA damage induction [94]. It was also found that epicatechin protects against DNA damage induced by N-nitrosodibutylamine (NDBA) and N-nitrosopiperidine (NPIP) in human hepatocarcinoma cells [95]. The epicatechin myricetin activates nonhomologous end-joining DNA double-strand break repair in human small intestinal cells [96]. Therefore, flavonoids can reduce DNA damage and enhance the DNA repair ability of cells, thereby reducing the accumulation of unrepaired damage.

Oxidative damage is believed to play a key role in pathological processes related to aging and age-related diseases, and its underlying biochemical mechanisms have been elucidated in detail [2,97]. Antioxidant capacity is an important activity of flavonoids. In APRE-19 cells, the solid dispersion of apigenin upregulates the expression of antioxidant enzymes and upregulates autophagy through the Nrf2 pathway, thereby inhibiting retinal oxidative damage [98]. In a rat natural aging model, fisetin significantly reduces pro-oxidants and increases the level of antioxidants to combat oxidative stress induced by aging [99]. Dihydromyricetin can reduce the oxidative damage of human umbilical vein endothelial cells induced by sodium nitroprusside by activating the PI3K/Akt/FoxO3a signaling pathway [100]. Nobiletin attenuates palmitate-induced ROS and mitochondrial dysfunction in cultured alpha mouse liver 12 cells [101]. In addition, naringenin [102], luteolin [103], genistein [104], kaempferol [105], and quercetin [106] have all been observed to inhibit oxidative damage in a variety of ways. Therefore, flavonoids may eliminate oxidative damage in senescent cells and help cells to overcome aging and aging-related diseases.

Flavonoids are also involved in the process of reducing and removing protein damage. Epicatechin upregulates eukaryotic translation elongation Factor 1A (eEF1A) through the 67 kDa laminin receptor [107]. Fisetin treatment of preadipocytes reduced the phosphorylation of the 70 kDa ribosomal protein S6 kinase 1 (S6K1). Nobiletin significantly blocked the activation of Akt/mTOR signaling and significantly inhibited the phosphorylation of S6K1 and eukaryotic translation initiation factor 4E-binding protein 1 (4EBP1) [108]. Phosphorylated S6K targets eIF4B and ribosomal protein S6 (RPS6). At the same time, 4EBP binds to eukaryotic initiation factor 4E (eIF4E) at the eIF4E–eIF4G interaction interface to prevent it from forming the translation initiation complex [109], thereby affecting translation fidelity.

Quercetin can specifically silence the expression level of HSP70. Previous studies have shown that HSP90 inhibitors have senolytic activity [110]. Luteolin can alleviate psoriasis’s pathological changes and symptoms by reversing the effects of IFN-γ and HSP90 expression and exosomal secretion, regulating the proportion of immune cells and inhibiting psoriasis. Myricetin interferes with the binding of HSP90β and TGF-β receptor II, thereby preventing fibroblast activation. This indicates that flavonoids can also regulate the activity of chaperone molecules. Proteasome activity and autophagy are important parts of protein quality control and a meaningful way to eliminate damaged proteins. Myricetin is reported to eliminate neurodegenerative protein aggregation by upregulating the proteasome degradation mechanism [111]. Quercetin and rutin are positive regulators of the Nrf2 transcription factor, which enhances the expression of proteasome catalytic subunits in neurons [112]. Fisetin promotes the survival of nerve cells by enhancing the activity of the proteasome when trophic factors are withdrawn [113]. Related reports indicate that all flavonoids listed in Table 2 are involved in the regulation of autophagy levels [114,115,116,117,118,119,120,121]. In summary, flavonoids can enhance protein quality control in various ways, thereby reducing protein damage.

The removal of lipofuscin in cells results in reduced lipid damage, which is often accompanied by improved aging-related pathology. Anti-aging studies on flavonoids have shown that they also can minimize lipofuscin in cells. Several studies have shown that kaempferol, myricetin, naringin, and quercetin can significantly reduce lipofuscin accumulation in *C. elegans*, a marker of aging [58,122,123]. However, rutin and fisetin, which also prolong the lifespan of nematodes, cannot delay the accumulation of lipofuscin in cells [122,123]. Quercetin can also inhibit the development of lipofuscin-related autofluorescence in senescent cells [124]. In addition, the accumulation of lipofuscin is closely related to mitochondrial function and lipid metabolism [30]. Flavonoids regulate mitochondrial function; for example, luteolin increases mitochondrial respiration in primary neurons [125]. Flavonoids can reduce lipofuscin in cells and affect the related processes of lipofuscin production.

Collectively, flavonoids effectively reduce the damage of DNA, protein, and lipid macromolecules by reducing the insults of damage. At the same time, they can improve the ability of damage repair or clearance, thereby significantly reducing the rate of unrepaired damage accumulating in cells. Due to the important role of unrepaired damage in inducing cell senescence, cells or tissues can benefit from the anti-damage effects of flavonoids.

## 5. Clinical Applications of Flavonoid on Aging

As mentioned above, preclinical results have shown that flavonoids have beneficial effects in attenuating cell senescence. These beneficial effects of flavonoids could apply to humans and are currently being tested in clinical trials (Table 2). Senolytic quercetin plus dasatinib and fisetin have been used in the clinical treatment of osteoporosis, diabetic kidney disease, Alzheimer’s disease, and other aging-related diseases. It is worth noting that fisetin has been included in several clinical studies to improve the health of the elderly population with COVID-19. In addition, two clinical studies on the efficacy of fisetin in reducing frailty and inflammation markers, insulin resistance, and bone resorption in the elderly are also being recruited for. Other flavonoid- and aging-related clinical research is rarely carried out, and only genistein has completed clinical trials in Alzheimer’s disease and metabolic syndrome. Rutin and vitamin C have also been included in clinical studies for type 2 diabetes mellitus.

In summary, although senotherapy consisting of flavonoids has been included in clinical research on aging states and aging-related diseases, there are no definite experimental results yet. The safety and possible side effects of the long-term use of flavonoids as anti-aging drugs also need to be considered in future clinical research.

## 6. Concluding Remarks

Flavonoids can be used as senolytic drugs to remove senescent cells in tissues, improve aging-related physiological phenotypes, and act as “senomorphics” to inhibit inflammation and immune senescence caused by SASPs. In recent years, many flavonoids have also emerged as anti-aging agents. For example, nobiletin can have an anti-aging effect by inhibiting the ROR protein from regulating the circadian rhythm cycle. At the same time, many studies have shown that flavonoids can eliminate the damage of macromolecules in cells, improve the ability of DNA repair, and improve the level of protein quality control, thereby reducing cell senescence and improving systemic aging. Due to the central role of macromolecular damage in aging, flavonoid therapy will be an effective anti-aging strategy. In addition, the flavonoids quercetin and fisetin have been included in a variety of clinical studies on aging-related states. These preclinical and clinical studies on flavonoids to delay aging provide an important data basis for applying flavonoids in treating aging and aging-related diseases.

Although many studies have revealed the anti-aging beneficial effects of flavonoids, attention should be given to the fact that the flavonoids currently used have unclear toxicity and side effects of long-term continuous use, low solubility, rapid metabolism, and poor absorption of dietary flavonoids in the gastrointestinal tract, which hinder their pharmacological potential. Fortunately, the use of nanoparticle-based formulations of flavonoids can significantly improve the pharmacology of flavonoids [126]. We have reason to believe that with more research discoveries, natural product flavonoids will inevitably enrich our anti-aging tool library more powerfully and provide alternative options for the development and application of clinical anti-aging drugs.

## Figures and Tables

**Table 1:** Overview of the modulatory anti-aging effect of flavonoids and related mechanisms.

Flavonoids	Structure	Targets	Activity	Lifespan Extension	Reference
Senolytic					
Quercetin	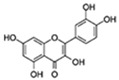	Numerous (including PI3K)	↓Senescent human cells in vitro; ↓SASP and hepatic steatosis in mice; ↓AD in mice; ↓Insulin resistance in obese mice; ↓Physical dysfunction in human IPF patients; ↓Anxiety in obese mice; ↑Exercise capacity; ↑Renal function in obese mice	15–60% lifespan in mice and *C. elegans*	[6,46,47]
Fisetin	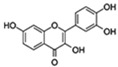	PI3K/AKT/mTOR	↓Senescent human cells in vitro; ↓Senescent cells and SASP in progeroid and aged mice in vivo; ↓Age-related pathology; ↑Lifespan of wild type in aged mice	~13% in mice, 23% in *Drosophila*, and 55% in *Saccharomyces cerevisiae*	[9,48]
Luteolin	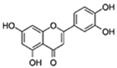	ROS, PGE_2_, COX2	↓Senescent human cells and SASP in vitro	No data	[10]
Senomorphic					
Apigenin	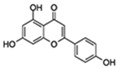	NF-κB p65 subunit IκB	↓SASP in fibroblasts; ↓SASP in the kidney of age rats; ↓Age-related skeletal muscle atrophy	No data	[49]
Kaempferol	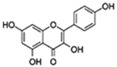	IRAK1/IkBα/NF-κB p65	↓SASP in fibroblasts; ↓ROS; ↓AGEs	No data	[50]
Others with antisenescence activity					
4,4′Dimethoxychalcone	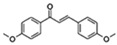	Autophagy GATA transcription factors	↓Cell senescence; ↑Health span	Approximately 20% increase in *Drosophila* and *C. elegans*	[51]
Naringenin	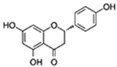	SIRT1/LKB1/PGC1α/NF-κB	↓Cardiac markers of aging-induced damage; ↓ROS	22.62% increase in females of *Drosophila*	[52,53,54]
Genistein	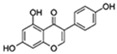	NF-κB p38	↓Proinflammatory genes expression; ↓Cell senescence; ↑Parameters of cognition in AD	No data	[55,56,57]
Myricetin	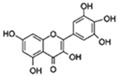	FOXO SIRT1/PGC-1α	↓HMW-Aβ-induced neurotoxicity; ↑Mitochondrial function	32.9% in increase *C. elegans*	[58,59]
Dihydromyricetin	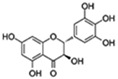	FOXO/ AOP Autophagy	↓Oxidative stress and inflammation-related senescence; ↓Gut dysfunction; ↑Motor and cognitive behavior	16.07% increase in *Drosophila*	[60,61,62]
Epicatechin	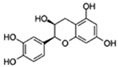	Autophagy	↓Cell senescence; ↓Skeletal muscle degeneration; ↑Brain function	7.1% increase in *C. elegans*	[63,64,65]
Nobiletin	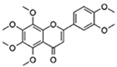	Target RORs	↓ROS; ↓Metabolic disease; ↑Circadian rhythms	1 month longer at median lifespan in mouse	[66,67]
Rutin	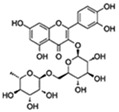	Insulin/IGF1 Autophagy	↓ROS and proinflammatory cytokines (TNF-α and IL-1β); ↓Aging-related metabolic dysfunction; ↑ATGs, Foxo	32% increase HFD *Drosophila*	[68,69,70]
Hesperidin	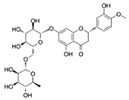	Nrf2	↓ROS; ↑Activity of antioxidant enzymes	Extends the repilicative lifespan of the yeast	[71,72]
Theaflavin	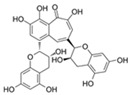	Nrf2	↓Stem cell senescence; ↑Intestinal homeostasis	Lifespan increase in *Drosophila*	[73,74]

SASP: senescence-related secretion phenotype; AD: Alzheimer’s disease; ATGs: autophagy-related genes; HFD: high-fat diet, ROR: retinoid acid receptor-related orphan receptor, AGEs: advanced glycation end products. ROS: reactive oxygen free radicals; SOD: superoxide dismutase; CAT: catalase; “↑” increased; “↓” decreased.

**Table 2:** Human clinical trials focusing on aging.

Flavonoid Therapy	Indication	Dose and Duration	Trial
Quercetin (Q) + Dasatinib (D)	Alzheimer’s disease	Q (1000 mg/day) + D (100 mg/day) administered orally for 2 consecutive days every 15 days (2 days on drug, 13 days off) for 6 cycles	NCT04785300
		Intermittent D + Q administered for 2 days on/14 days off for 12 weeks (6 cycles)	NCT04063124
	Age-related osteoporosis	D (100 mg/2 days) plus Q (1000 mg/day last for 3 days) taken orally on an intermittent schedule (starting every 28 days) over 20 weeks, resulting in five total dosing periods throughout the entire intervention	NCT04313634
	Accelerated-ageing-like state post bone marrow transplantation	Q (1000 mg/day) + D (100 mg/day) administered orally for 3 consecutive days	NCT02652052
	Diabetic kidney disease	Q (1000 mg/day) + D (100 mg/day) administered orally for 3 consecutive days	NCT02848131
	Epigenetic aging	500 mg Q and 50 mg D oral capsules on Monday, Tuesday, and Wednesday (3 days in a row) for 6 months	NCT04946383
Fisetin	Age-related osteoporosis	20 mg/kg/day for three consecutive days, taken orally on an intermittent schedule (starting every 28 days) over 20 weeks, resulting in five total dosing periods throughout the entire intervention	NCT04313634
	Elderly syndrome	20 mg/kg/day, orally for 2 consecutive days	NCT03675724
	Elderly syndrome in old women	20/mg/kg/day, orally for 2 consecutive days, for 2 consecutive months	NCT03430037
	Osteoarthritis	Administered orally at 20 mg/kg for 2 consecutive days, followed by 28 days off, then 2 more consecutive days	NCT04210986
		Oral fisetin 20 mg/kg taken for 10 days total	NCT04815902
	Diabetic and chronic kidney disease	20 mg/kg/day, orally for 2 consecutive days	NCT03325322
	COVID-19 in hospitalized patients	20 mg/kg/day, orally for 2 consecutive days	NCT04476953
	COVID-19 in outpatients	20 mg/kg/day oral for 4 days	NCT04771611
	Coronavirus disease 2019 (COVID-19) in nursing home patients	20 mg/kg/day, orally for 2 consecutive days	NCT04537299
Genistein	Alzheimer’s disease	60 mg of genistein BID for 360 days	NCT01982578
	Metabolic syndrome	Genistein capsules of 25 mg each, 50 mg/day	NCT04105023
Rutin	Type 2 diabetes mellitus	Rutin 60 mg in combination with vitamin C 160 mg three times daily in addition to usual antidiabetic treatment for 8 weeks.	NCT03437902
